# Supplementary material for: Pharmacokinetics of Levodopa and 3-O-Methyldopa in Parkinsonian Patients Treated with Levodopa and Ropinirole and in Patients with Motor Complications
Source: Pharmaceutics. 2021 Sep 3;13(9):1395. doi: 10.3390/pharmaceutics13091395 (PMC8472364; doi:10.3390/pharmaceutics13091395)
Supplement: Supplementary file 1 [file pharmaceutics-13-01395-s001.zip › pharmaceutics-1296164 - sp for check.pdf]

pharmaceutics

MDPI

Supplementary Materials: Pharmacokinetics of Levodopa and 3-O-Methyldopa in Parkinsonian Patients Treated with Levodopa and Ropinirole and in Patients with Motor Complications

Urszula Adamiak-Giera *, Wojciech Jawień, Anna Pierzchlińska, Monika Białecka, Jan Dariusz Kobierski, Tomasz Janus and Barbara Gawrońska-Szklarz *

Content

Material and Method 1

Calculation of the secondary individual PK parameters 3

Results 3

Measured concentrations 3

Estimated parameters of the population model 4

Model diagnostics 6

Discussion 10

Model diagnostics 10

Properties of the final population model 10

References 10

Material and Method

The assumed PK model (Figure 1[1]) can be described by the following system of differential equations:

$$\frac{dA_{Ld}}{dt} = -(k_{10} + k_{12})A_{Ld} + k_a A_{dep}$$

$$\frac{dA_{3OMD}}{dt} = k_{12}A_{Ld} - k_{20}A_{3OMD}$$

$$\frac{dA_{dep}}{dt} = -k_a A_{dep}$$

A_{dep} and A_{Ld} are amounts of L-dopa in the depot and central compartments, respectively. A_{3OMD} is an amount of 3-OMD in the central compartment.

The definition of this model expressed in the model description language MlxTran [1] is shown in Figure S1.

[1] The numbers of figures, references etc. without the prefix S refer to the main text; those with S prefix refer to the present document – supplementary materials.

```
; A PK model for L-DOPA (LD) and its metabolite,
; L-3-O-Methyldopa (L3OMD)
; Formula weights
; LD 197.19
; L3OMD 211.22
;
DESCRIPTION:
L-DOPA & L-3-OMD metabolite system, extravascular administration, 1 compartment

INPUT:
parameter = {Tlag, ka, k10, k12, k20, VC} ; VC is a scale parameter, not simply Vd. It contains
                                          ; F as well.
PK:

compartment(amount=CLD,cmt=1)  ; Central compartment LD
compartment(amount=C3OMD,cmt=2); Central compartment 3OMD

oral(Tlag, ka, cmt=1)

EQUATION:
LDtoL3OMD = 197.19 / 211.22

ddt_CLD = -(k10+k12)*CLD
ddt_C3OMD = k12*CLD*LDtoL3OMD - k20*C3OMD

O1 = CLD / VC
O2 = C3OMD / VC

OUTPUT:
output = {O1,O2}
```

Figure S1. Description of the pharmacokinetic model in the MlxTran [1] language.

With the aid of the Monolix [2] software release 2019R1 (Lixoft, Antony, France) the population pharmacokinetic model has been implemented for the drug-metabolite system. The pharmacokinetic parameters of the model were estimated and their dependence on covariates evaluated. Continuous variables: age, height, weight, creatinine concentration, ropinirole concentration after 2 h after its administration (if any) as well as categorized variables: sex, motor complications and ropinirole therapy were considered as covariates.

Taking advantage of the automatic model selection feature available in 2019R1 release the optimal set of covariates and an optimal model of the residual error were determined. The selection algorithm is based mainly on AIC criterion [2]. The optimal set of covariates contained: sex and ropinirole concentration (2h after the administration) as predictors for the parameter V/F, age for k_{10}, weight and motor complications for T_{lag}, ropinirole therapy for k_{20}.

The final population PK model equations are as follows:

$$\log V/F = \log(V/F)_{pop} + \text{sex} \times \beta_{V/F\,sex} + \text{rop2h} \times \beta_{V/F\,rop2h} + \eta_{V/F}$$

$$\log k_{10} = \log k_{10\,pop} + (\text{age} - \text{median\_age}) \times \beta_{k10\,c\_age} + \eta_{k10}$$

$$\log T_{lag} = \log T_{lag\,pop} + (\text{weight} - \text{median}_{\text{wt}}) \times \beta_{Tlag\,c_{wt}} + \text{mot\_comp} \times \beta_{Tlag\,mc} + \eta_{Tlag}$$

$$\log k_{20} = \log k_{20\,pop} + \text{rop\_th} \times \beta_{k20\,rop\_th} + \eta_{k20}$$

$$\log k_a = \log k_{a\,pop} + \eta_{ka}$$

Population values of PK parameters have an index *pop*. Fixed effect parameters are denoted by β with appropriate indices, while for random effects symbols $\eta_{.}$ are used. It is assumed that the vector of random effects $\boldsymbol{\eta}$ has a multivariate normal distribution with zero mean and a covariance matrix $\boldsymbol{\Omega}$; elements of this matrix will be referred to by $\omega_{..}$ in Results section. Symbols 'sex', 'weight' and 'age' have an obvious meaning; medians of body mass and age in the investigated group have symbols 'median_wt' and 'median_age', respectively. Motor complications are denoted as

'mot_comp', ropinirole therapy as 'rop_th' and ropinirole concentration after 2h by 'rop2h'. For all random effects the log-normal distribution was assumed. For residual errors ε the following models were adopted:

L-dopa – type 2 combined model: $\varepsilon_{L-dopa} = \left(b_1^2 C_{th\,L-dopa}^2 + a_1^2\right)^{1/2} \times \varepsilon$

3-OMD – type 1 combined model: $\varepsilon_{3OMD} = (b_2 C_{th\,3OMD} + a_2) \times \varepsilon$

a_1, a_2, b_1, b_2 are symbols of error model parameters. $C_{th...}$ are theoretical (predicted by the PK model) concentration values, and ε is a random variable that undergoes the standard Gaussian distribution.

Calculation of the Secondary Individual PK Parameters

Based on the estimated individual primary PK parameters the secondary parameters with more direct clinical meaning were calculated using following equations.

For L-dopa:

$$CL/F = (k_{10} + k_{12}) \times V/F$$

$$AUC = \frac{D}{CL/F}$$

$$MRT = \frac{V/F}{CL/F}$$

$$t_{\max} = T_{lag} + \frac{\ln\left(\frac{k_{10} + k_{12}}{k_a}\right)}{k_{10} + k_{12} - k_a}$$

$$C_{\max} = \frac{D k_a}{V/F} \times \frac{\exp(-k_a \tau) - \exp[-(k_{10} + k_{12})\tau]}{k_{10} + k_{12} - k_a}$$

where $\tau = t_{\max} - T_{\text{lag}}$

for 3-OMD:

$$MET = T_{lag} + \frac{1}{k_{10} + k_{12}} + \frac{1}{k_{21}} + \frac{1}{k_a}$$

$$AUC_{3-\text{OMD}} = \frac{D}{V/F} \times \frac{k_{12}}{k_{20}(k_{10} + k_{12})}$$

The equation for $t_{\max 3-\text{OMD}}$ was obtained with the aid of Wolfram Mathematica 12.1 (Wolfram Research, Champaign, IL, USA) [3]. It reads:

$$\begin{aligned} & (k_{10} + k_{12})(k_{20} - k_a)\exp[-(k_{10} + k_{12})t_{\max 3-\text{OMD}}] \\ + \quad & k_a(k_{10} + k_{12} - k_{20})\exp(-k_a t_{\max 3-\text{OMD}}) \\ - \quad & k_{20}(k_{10} + k_{12} - k_a)\exp(-k_{20} t_{\max 3-\text{OMD}}) = 0 \end{aligned}$$

It appears that there is no closed-form solution to this equation. Because of that Wolfram Mathematica was also used to numerically solve it for $t_{\max 3-\text{OMD}}$. The obtained solution was then used to calculate $C_{\max 3-OMD}$ from the model equation.

Results

Measured Concentrations

All measured plasma L-dopa and 3-OMD concentrations in patients under the study are depicted in Figures S2 and S3.

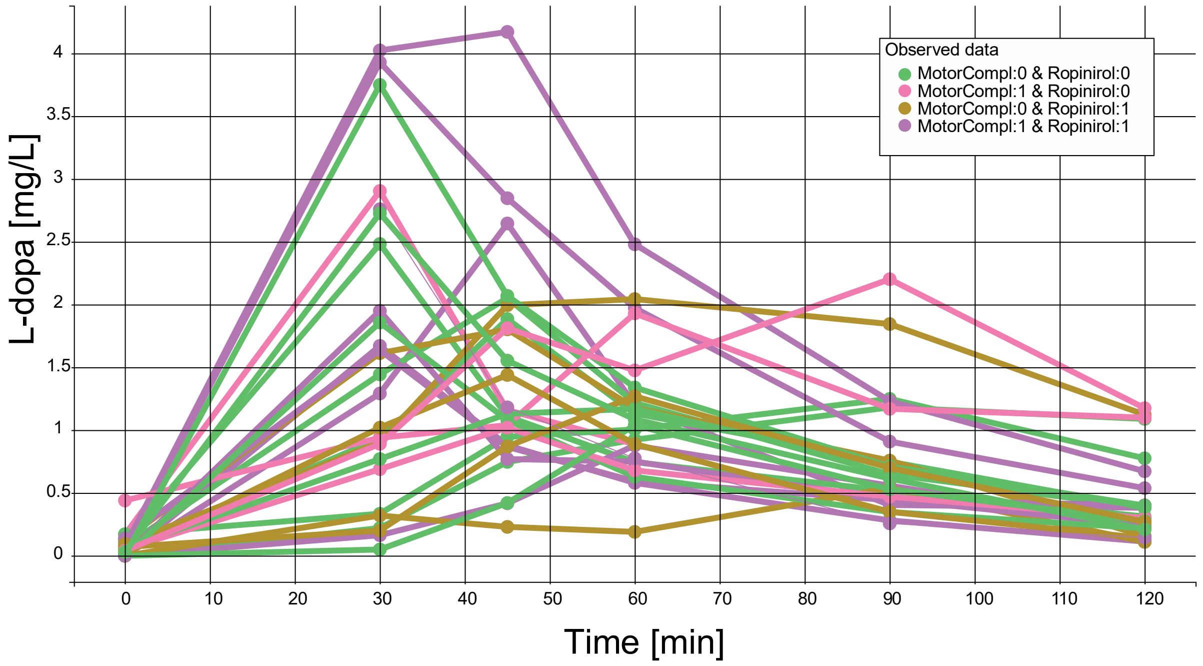

Figure S2. Spaghetti plot of all measured L-dopa concentrations.

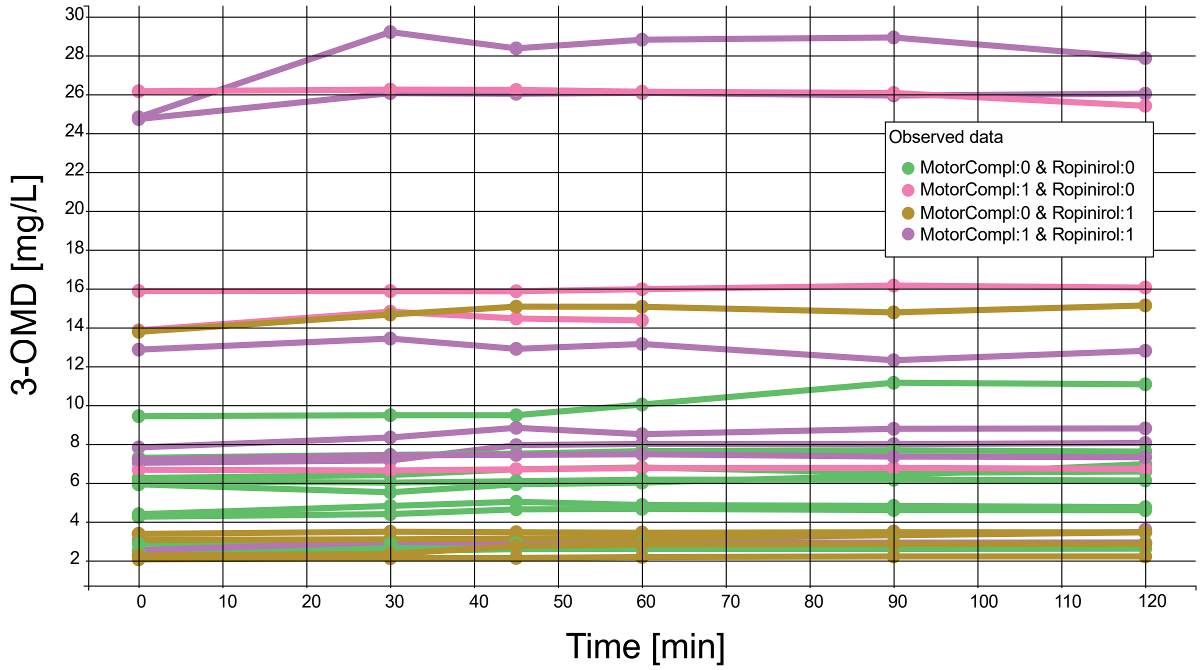

Figure S3. Spaghetti plot of all measured 3-OMD concentrations.

Estimated Parameters of the Population Model

The estimated parameters of the population model are compiled in Table S1.

Table S1. Estimated parameters of the population model.

Parameter	Value	S.E.	R.S.E. (%)
**Fixed Effects**			
$T_{lag\,pop}$ [min]	17.9	2.5	13.9
$\beta_{Tlag\,c\_wt}$ [min/kg]	0.0135	0.00544	40.4
$\beta_{Tlag\,mc}$ [min]	−4.99	0.277	5.55
$k_{a\,pop}$ [min^{-1}]	0.0903	0.0372	41.3
$k_{10\,pop}$ [min^{-1}]	0.0207	0.00178	8.6
$\beta_{k10\,c\_age}$ [min^{-1}/yr]	−0.0191	0.00704	36.8
$k_{12\,pop}$ [min^{-1}]	0.00266	0.000356	13.4
$k_{20\,pop}$ [min^{-1}]	0.000159	2.63×10^{-5}	16.6
$\beta_{k20\,rop\_th}$ [min^{-1}]	0.334	0.142	42.6
$(V/F)_{pop}$ [L]	43.1	3.34	7.75
$\beta_{V/F\,rop2h}$ [L^2/mg]	−0.0807	0.0202	25.1
$\beta_{V/F\,sex}$ [L]	−0.611	0.0946	15.5
**Standard Deviation of the Random Effects**			
ω_{Tlag}	0.123	0.0527	43
ω_{ka}	1.83	0.3	16.4
ω_{k10}	0.292	0.0546	18.7
ω_{k12}	0.248	0.0864	34.8
ω_{k20}	0.167	0.131	78.3
$\omega_{V/F}$	0.023	0.286	**1.24×10^3**
γ_{k12}	0.00129	0.108	**8.42×10^3**
**Error Model Parameters**			
a_1 [mg/L]	0.0809	0.00893	11
b_1	0.237	0.022	9.27
a_2 [mg/L]	0.0318	0.0129	40.5
b_2	0.0309	0.000701	2.27

S.E. – standard error, R.S.E. – relative standard error

The distribution of individual parameters can be seen in Figure S4.

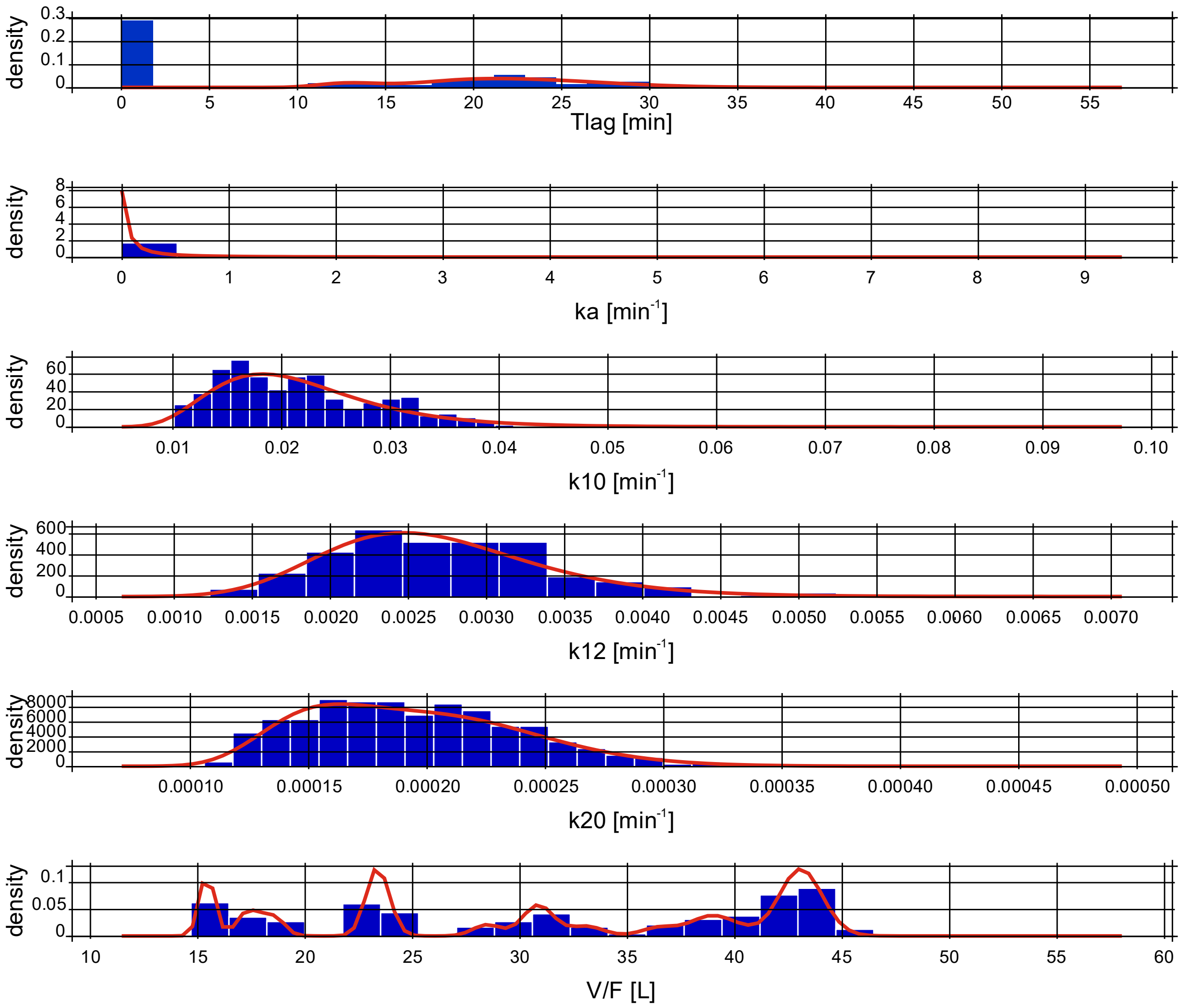

Figure S4. Empirical (bars) and theoretical (line) distributions of individual pharmacokinetic parameters.

Model Diagnostics

Plots of relationship between observed vs predicted concentrations for both investigated compounds in individual patients are shown in Figures S5 and S6. In order to verify a model adequacy, NPDE scatterplots [4] were prepared (Figures S7 and S8). In addition, normality plots for NPDE are shown (Figures S9 a and b). Model diagnostics also included visual predictive check plots [2] for L-dopa (Figure S10) and 3-OMD (Figure S11).

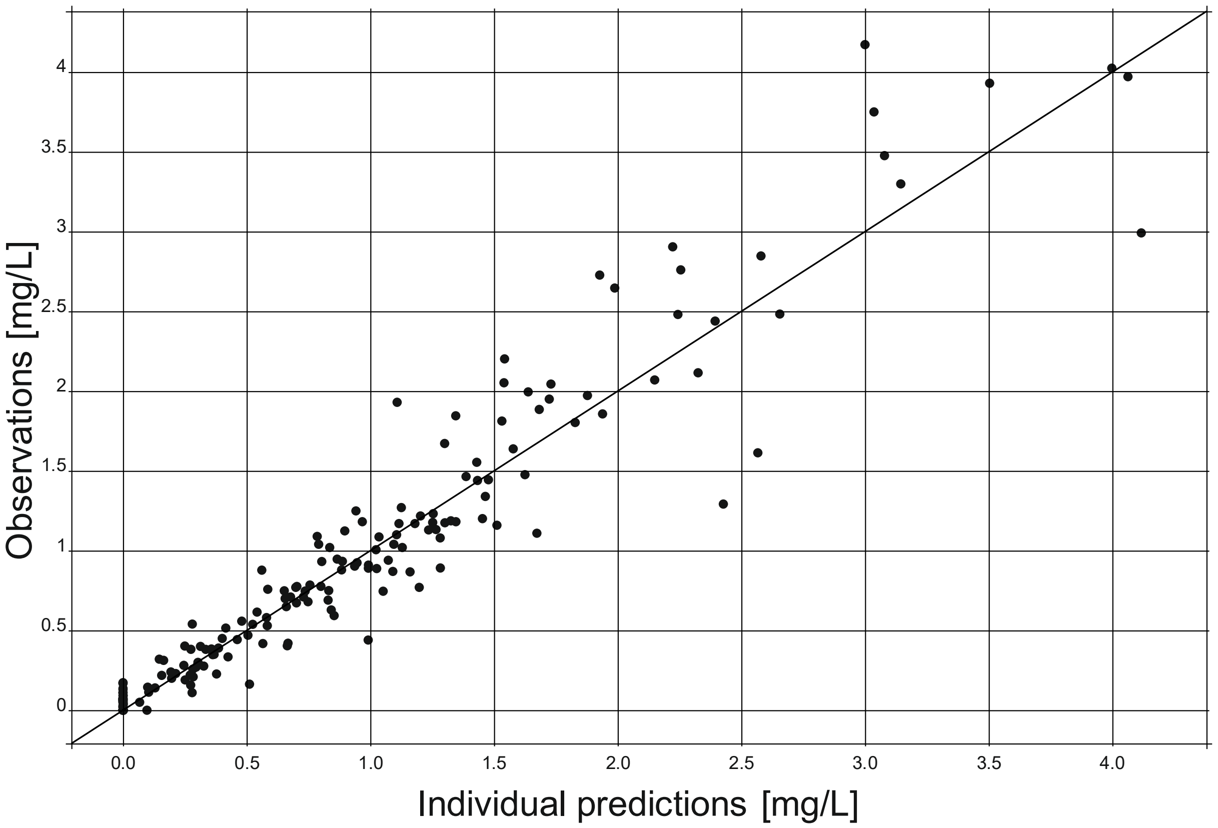

Figure S5. Plots of relationship between observed vs predicted concentrations for L-dopa in individual patients.

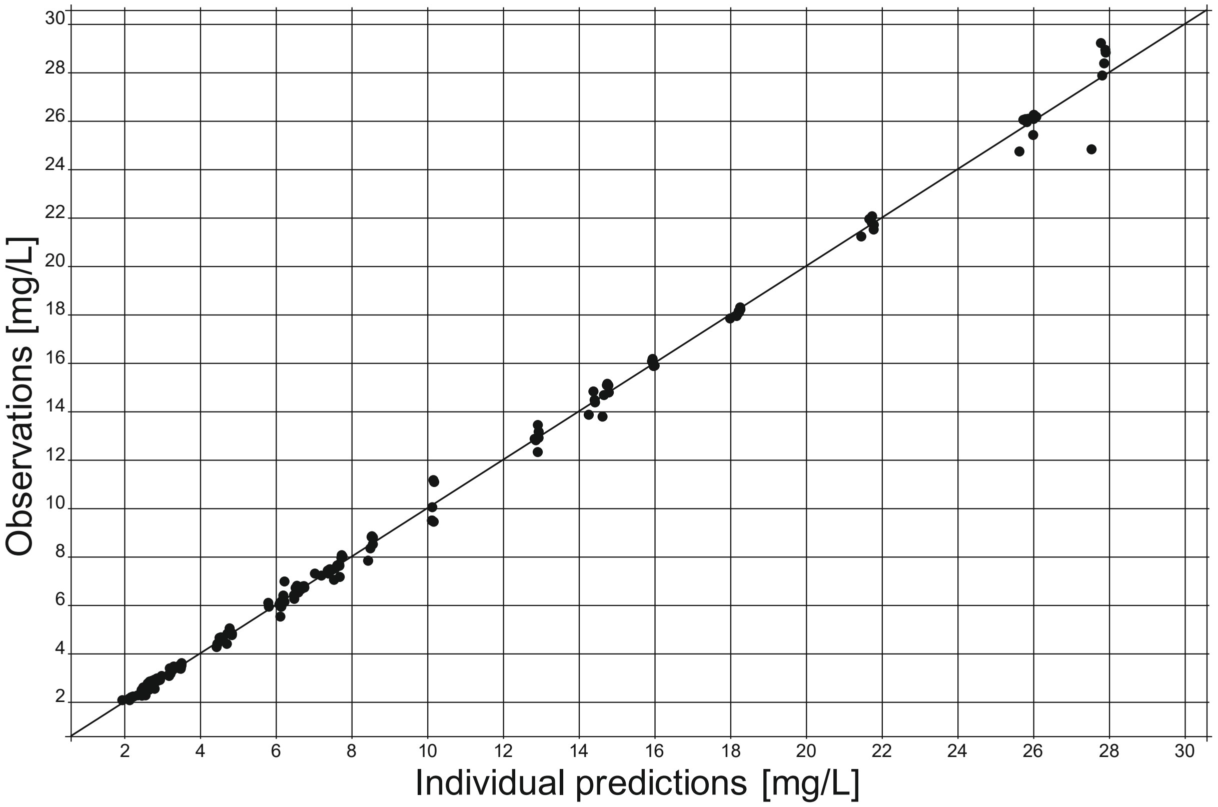

Figure S6. Plots of relationship between observed vs predicted concentrations for 3-OMD in individual patients.

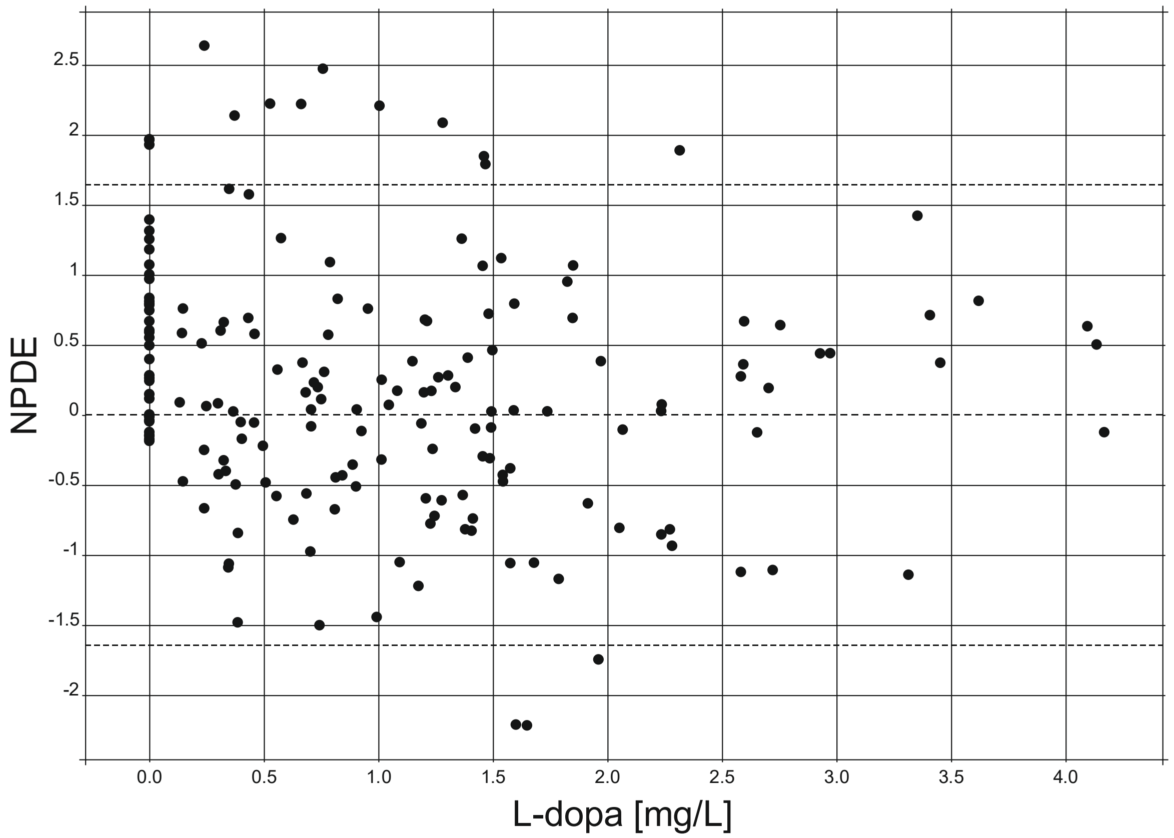

Figure S7. NPDE scatterplot for L-dopa.

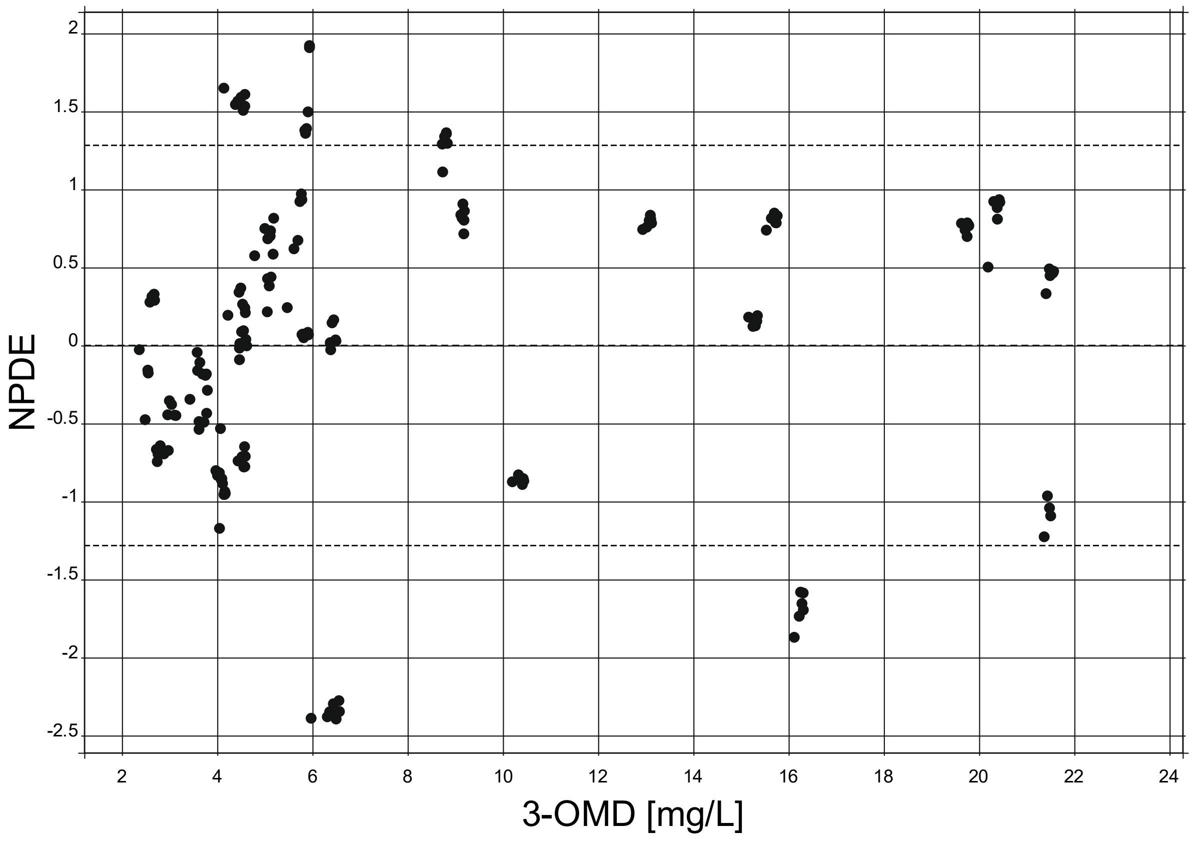

Figure S8. NPDE scatterplot for 3-OMD.

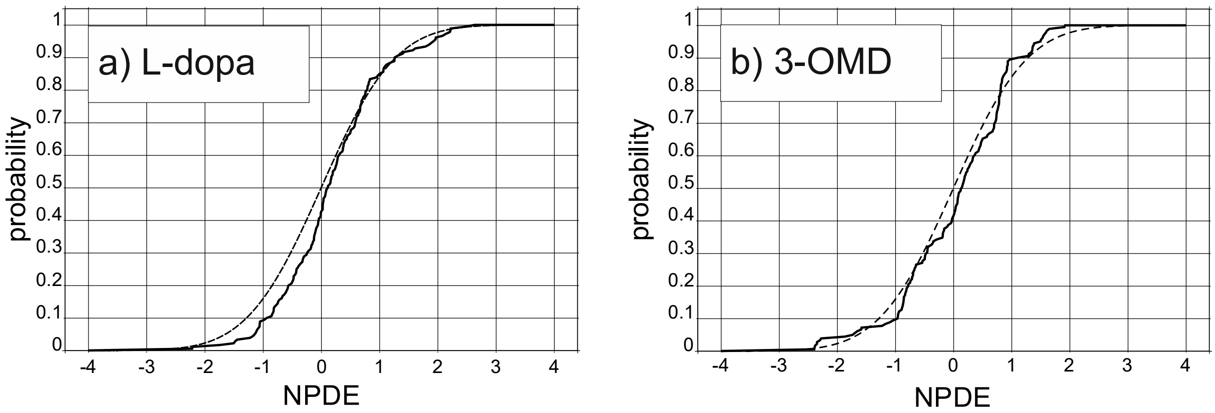

Figure S9. NPDE normality plots for a) L-dopa and b) 3-OMD.

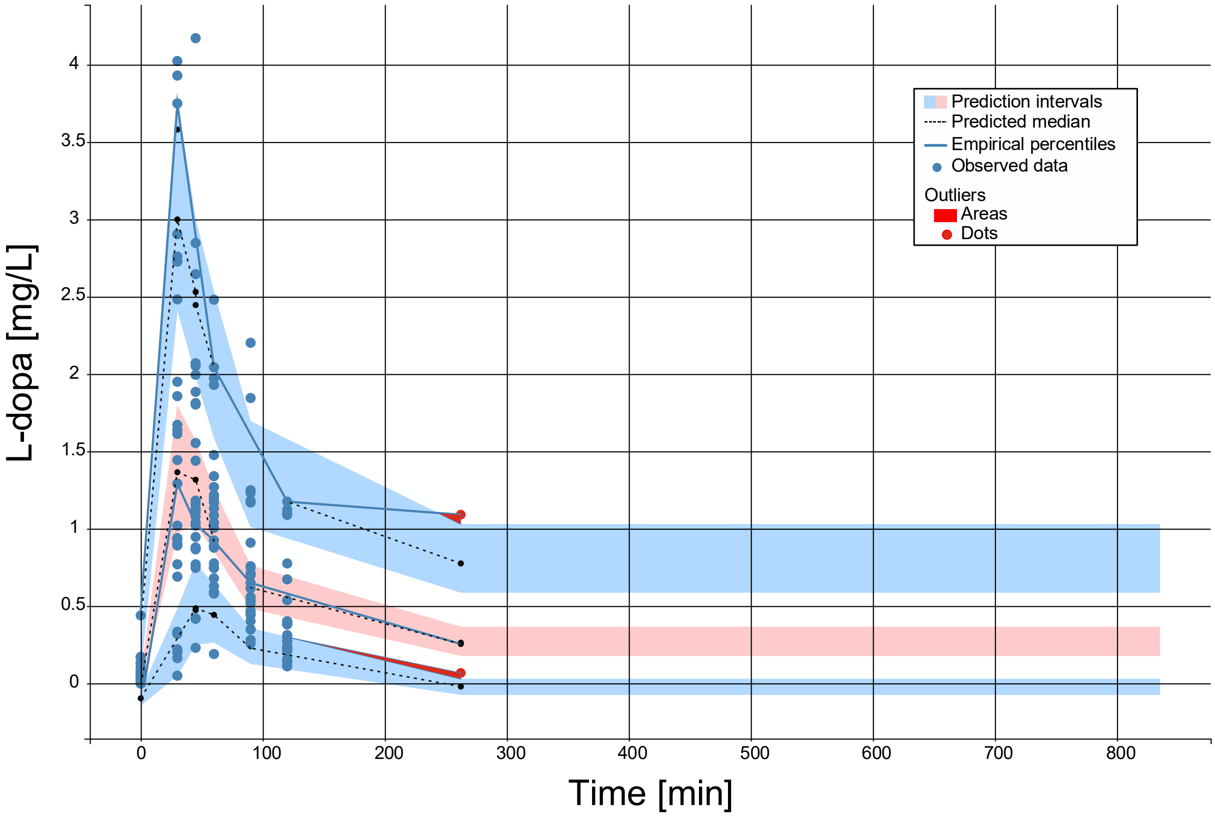

Figure S10. Visual predictive check plot for L-dopa.

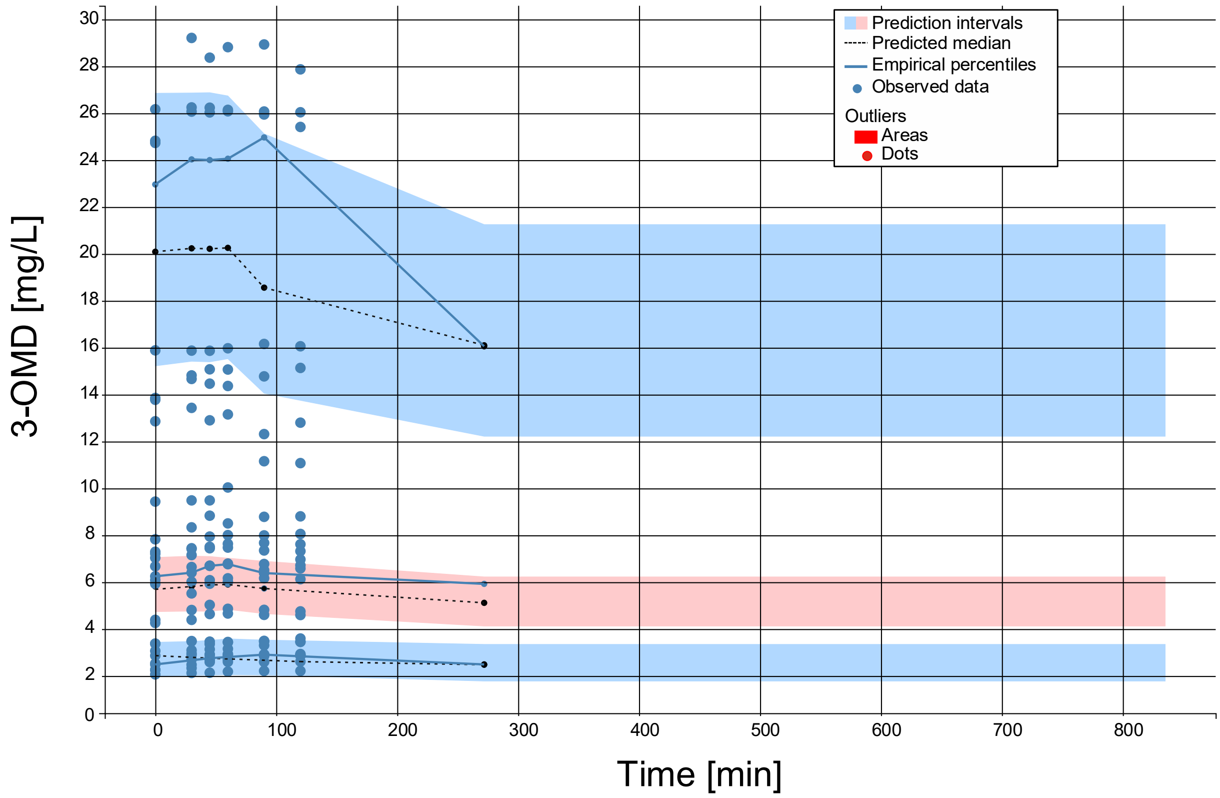

Figure S11. Visual predictive check plot for 3-OMD.

Discussion

Model Diagnostics

NPDE scatterplot for L-dopa does not show regular patterns. Only at time 0 model predictions were very close to 0, while in a few patients remarkably non-zero L-dopa concentrations were observed immediately prior to the test dose administration. For 3-OMD there are clusters of data visible, however these clusters seem to be randomly distributed. Normality plots indicate a good agreement of empirical distribution with the standard normal distribution. Visual predictive check displayed only two small outlier regions for L-dopa. No problems were detected on visual predictive check plot for 3-OMD. Therefore there are no clear symptoms of model misspecification.

Properties of the Final Population Model

The lag time distribution was found bimodal: very short times correspond to patients with motor complications, and times of order of 20 minutes were observed in patients without that impairment. Another factor which proved to have an impact on the lag time was body weight: this time parameter increases slightly with patient's weight.

First order absorption constant has a sharp maximum at 0.09 min^{-1} and a long right tail. Rather high relative standard error of this estimate (41%) reflects common problems in estimation of this parameter unless the sampling in the absorption phase is very dense, what is normally precluded because of ethical reasons. No covariate demonstrated statistically significant impact on this absorption parameter.

Remaining first order speed constants of the PK model: k_{10}, k_{20} and k_{12} have more regular distributions. The elimination constant of L-dopa (on routes other than metabolism to 3-OMD), k_{10}, decreases with the patient's age. The elimination constant of 3-OMD, k_{20} had lower expectance in these patients who were co-medicated with ropinirole.

The scale parameter (V/F) proved to be lower in females. The regression-type dependence on concentration of ropinirole observed 2 h after its administration was also observed: V/F decreases with that concentration.

Random effect parameter that appears to be poorly estimated is related to *V/F*. It means that dispersion of V/F across the population could not be reliably estimated based on available data.

References

1. Mlxtran User Guide, *Lixoft*, 2019. Available online: http://mlxtran.lixoft.com/mlxtran-user-guide/ (accessed on 30 April 2019).
2. Monolix Documentation Version 2019, *Lixoft*, 2019. Available online: http://monolix.lixoft.com/single-page/ (accessed on 30

April 2019).
3. Wollfram Research, “Mathematica.” Wolfram Research, Inc., Champaign IL, 2020. Available online: https://www.wolfram.com/mathematica (accessed on 30 April 2019).
4. Brendel, K.; Comets, E.; Laffont, C.; Laveille, C.; Mentré, F. Metrics for external model evaluation with an application to the population pharmacokinetics of gliclazide. *Pharm. Res.* **2006**, *23*, 2036–2049, doi:10.1007/s11095-006-9067-5E.
